# The Physiology of Thought

**Published:** 1871-07

**Authors:** 


### Editorial.

### THE PHYSIOLOGY OF THOUGHT.

The mere title is startling. What do we know about thought itself, to say nothing of its physiology? And yet, mental philosophers make most of their failures through lack of attention to the physiological bearings of the subject.

In a well written article in this number of the REGISTER, the common sentiment is reasserted, in substance, that great thoughts emanate only from men physically strong. "A sound mind in a sound body" has a beautiful sound. For the ordinary purposes of society, such a man is very desirable; and it is best when such constitute the great majority of any community. But it is not from such men that the thoughts which move the world are obtained. Society is mainly molded, modified, controlled, by men far different.

A sound body assimilates carbon, hydrogen, nitrogen, oxygen, phosphorus, etc., in such proportion as to give uniformity of power to vital action. He holds a place similar to that of the general-purpose horse at the agricultural fair. If we raise but one style of horse, this is the one we want. But we must not expect him to run like an Arab, or draw the load of the Norman. So our general-purpose man is the best, if we must have but one; and if men were designed for hermits, it would be unwise to rear any other kind; but with man made for society, it is possibly better to have some variations.

A constitution which readily assimilates carbon, hydrogen and nitrogen, may give wonderful manifestations of what we usually call physical strength, provided it also appropriates enough of the other essential elements to keep up a reasonable balance; but it is not common for such to manifest great depth of thought. On the other hand, there may be a lack of ability to properly assimilate these elements. with an extraordinary power to appropriate phosphorus. Such a constitution will, if not greatly unbalanced, show great mental power, with a corresponding lack of physical force and energy. Who can not recall the memory of his schoolboy days, when the hero of the play-ground and the pet of the class-room were very different boys.

In all departments of society, unbalanced constitutions do the deepest thinking. Not that all such are good thinkers; but only those who overbuild the nervous tissue, by assimilating phosphorus, etc., till the muscular and secretory systems are over-topped.

Notable examples might be referred to: Among poets, Milton, blind; Hood, organic disease of heart, lungs, with nearly constant rheumatism; Cowper—too sad to tell all his ills; Pope, Kirke White. In the linguistic lore, what writer of English fails to feel the force of Lindley Murray, an invalid, bedfast for forty years,—all his writing done in bed. Did John Calvin, the invalid, make himself felt in the religious sentiment of the world? Scott, the cripple, wrote good poetry and readable novels. James Watt, the victim of neuralgia and sick-headache, made fair steam engines. Dr. Kane, traveling for his health, widened the field of knowledge. "Stonewall" Jackson, the sickly professor, was something of a fighter. Alex. Stevens, not strong enough to draw his last breath, but will die with an expiration, possesses some legal lore. Thad. Stevens, the scrofulous cripple, was something of a statesman.

But the time would fail me to tell of Johnson, of Webster, of Harvey, of Wilson, of Esop, of Cartright, of Prentiss, and others, whose thoughts we cherish as household jewels. Suffice it to say that in our own profession, for a dozen years at least, the leaders of thought, and the advance guard of professional progress, have been men with badly balanced and defective constitutions. When the physiology of thought is understood, this is not strange—not more strange than that the English racer can outrun the roadster. W.

